# Rapid Diagnosis of Diarrhea Caused by *Shigella sonnei* Using Dipsticks; Comparison of Rectal Swabs, Direct Stool and Stool Culture

**DOI:** 10.1371/journal.pone.0080267

**Published:** 2013-11-22

**Authors:** Claudia Duran, Faridabano Nato, Sylvie Dartevelle, Lan Nguyen Thi Phuong, Neelam Taneja, Marie Noëlle Ungeheuer, Guillermo Soza, Leslie Anderson, Dona Benadof, Agustín Zamorano, Tai The Diep, Truong Quang Nguyen, Vu Hoang Nguyen, Catherine Ottone, Evelyne Bégaud, Sapna Pahil, Valeria Prado, Philippe Sansonetti, Yves Germani

**Affiliations:** 1 Facultad de Medicina, Universidad de Chile, Santiago, Chile; 2 Institut Pasteur, Plate-Forme 5 - Production de Protéines recombinantes et d'Anticorps, Paris, France; 3 Pasteur Institute of Ho Chi Minh City, Department of Immunology & Microbiology, Ho Chi Minh City, Vietnam; 4 Department of Medical Microbiology, Postgraduate Institute of Medical Education and Research, Chandigarh, India; 5 Institut Pasteur, Plate-forme Investigation Clinique et Accès aux Ressources Biologiques, Paris, France; 6 Hospital Dr. Hernán Henríquez Temuco, Temuco, Chile; 7 Hospital Roberto del Río-Santiago, Región Metropolitana, Chili; 8 Paediatric Hospitals I, Ho Chi Minh City, Vietnam; 9 Institut Pasteur, Centre de Ressources Biologiques, Paris, France; 10 Institut Pasteur, Unité de Pathogénie Microbienne Moléculaire, INSERM U786, Paris, France; Institut Pasteur de Lille, France

## Abstract

**Background:**

We evaluated a dipstick test for rapid detection of *Shigella sonnei* on bacterial colonies, directly on stools and from rectal swabs because in actual field situations, most pathologic specimens for diagnosis correspond to stool samples or rectal swabs.

**Methodology/Principal Findings:**

The test is based on the detection of *S. sonnei* lipopolysaccharide (LPS) O-side chains using phase I-specific monoclonal antibodies coupled to gold particles, and displayed on a one-step immunochromatographic dipstick. A concentration as low as 5 ng/ml of LPS was detected in distilled water and in reconstituted stools in 6 minutes. This is the optimal time for lecture to avoid errors of interpretation. In distilled water and in reconstituted stools, an unequivocal positive reaction was obtained with 4 x 10^6^ CFU/ml of *S. sonnei*. The specificity was 100% when tested with a battery of *Shigella* and different unrelated strains. When tested on 342 rectal swabs in Chile, specificity (281/295) was 95.3% (95% CI: 92.9% - 97.7%) and sensitivity (47/47) was 100%. Stool cultures and the immunochromatographic test showed concordant results in 95.5 % of cases (328/342) in comparative studies. Positive and negative predictive values were 77% (95% CI: 65% - 86.5%) and 100% respectively. When tested on 219 stools in Chile, Vietnam, India and France, specificity (190/198) was 96% (95% CI 92%–98%) and sensitivity (21/21) was 100%. Stool cultures and the immunochromatographic test showed concordant results in 96.3 % of cases (211/219) in comparative studies. Positive and negative predictive values were 72.4% (95% CI 56.1%–88.6%) and 100 %, respectively.

**Conclusion:**

This one-step dipstick test performed well for diagnosis of *S. sonnei* both on stools and on rectal swabs. These data confirm a preliminary study done in Chile.

## Introduction

Acute infectious diarrhoea constitutes a significant cause of morbidity and infant mortality. Worldwide, it is estimated that 164.7 million people suffer from shigellosis annually: 163.2 million in developing countries and 1.5 million in developed countries [1,2]. *S. sonnei* is a causal agent of fever, nausea, stomach cramps, vomiting, and diarrheal disease, often complicated by the occurrence of a dysenteric syndrome. It accounts for most of the reported cases of shigellosis in developed areas and in emerging countries [3]. In the United States, 70% of shigellosis episodes are caused by *S. sonnei*. Historically, *S. sonnei* has been predominantly responsible for dysentery in developed countries; in recent years it has become the dominant serotype causing shigellosis in Asian countries [4] but is now emerging as a problem in the developing world, seeming to replace the more diverse *S. flexneri* in areas undergoing economic development and improvements in water quality [5].

One step immunochromatographic dipstick tests have been successfully developed at Institut Pasteur for cholera [6], *S. flexneri* 2a [7] and *S. dysenteriae* 1 [8]. Considering the potential impact this rapid diagnostic test have for the clinical management of the disease and for epidemiological studies, works are in progress at Institut Pasteur to develop rapid diagnostic tests able to detect several enteric pathogens. 

Rapid diagnosis of shigellosis is important because it allows to engage appropriate antimicrobial treatment that shortens the duration and severity of the illness and reduces microbial carriage, thus the spread of infection in the community. 

The dipstick method requires minimal technical skill, and the test can be read in about 10 minutes. Additionally, the dipsticks can be stored at room temperature in a humidity-proof plastic bag, making them easily transportable. 

In this effort, we evaluated the potential of a dipstick test to detect *S. sonnei* in stools and bacterial colonies.

Classical approaches have shown that *S. sonnei* is genetically conserved and clonal [5]. Unlike other *Shigella* species, all virulent *S. sonnei* strains comprise a single serotype which produces smooth colonies expressing a somatic antigen termed form I. This antigenic specificity corresponds to the O-side chains of the lipopolysaccharide layer, which are composed of disaccharide repeating subunits containing two unusual amino sugars, 2-amino-2-deoxy-L-altruronic acid (LAltNAcA) and 2-acetamido-4-amino-2,4,6-trideoxy-D-galactose (4-n-D-FucNAc) [9]. The genes encoding this O-polysaccharide are located on a 180-kb virulence plasmid [10], which also carries the invasion pathogenicity island [11]. Virulent form I colonies are typically unstable and upon replating convert at high frequency to rough colonies that still express the *Enterobacteriaceae* Ri lipopolysaccharide core, termed form II, due primarily to spontaneous loss of the large virulence plasmid and the ensuing loss of form I O antigen. 

The traditional identification by culture lacks sensitivity due to the low number of causative micro-organisms excreted, competition with commensal organisms, and deleterious changes in ambient temperature and pH during specimen transport [12, 13, 14.]. The detection is also frequently impaired by the use of antibiotics prior to specimen collection. The present work describes the second evaluation of this new test that addressed the issue of rapid diagnosis of *S. sonnei* diarrhoea and dysentery testing from bacterial cultures, stools and rectal swabs which is usually how the specimen is often collected or received from the field or from remote settings.

## Materials and Methods

### Ethics Statement

In Chile, India, France and Vietnam, written informed consents were obtained from all participants involved in the study. The study was approved by the Scientific and Ethical Committee of Pasteur Institute in Ho Chi Minh City (institutional review board included Nguyen Van Tam, MD; Nguyen Kim Dung, M sc; Nguyen Thi Nguyet Thu, M sc; Cao Minh Thang, M sc; Ho Thi Thien Ngan, MD). In Chile, the protocol, designed to set up new diagnostic methods for infectious diseases, has been approved by the Ethical Committee, School of Medicine, University of Chile, Santiago, Chile (review board included Manuel Oyarzun, Marianne Gaudlitz, Hugo Amigo, Leandr Biagini, Lucia Cifuentes, Nina Horwitz, Claus Jahn, Miguel O’Ryan, Julio Pallavicini). In India This study was approved by the Institute Ethics Committee of Postgraduate Institute of Medical education and Research (PGIMER) in Chandigarh (institutional review board included Girish Varshney, Jatinder Mohan, Kusum Joshi, Sudesh Prabhakar, Rajesh Kumar, Jai Dev Wig, Niranjan Khandelwal, Sanjay Jain, Sunil Arora, Nirmal Kumar Ganguly, Prem Kumar Palli, Arunaloke Chakrabarti). In France, fæces from healthy donors were supplied by the Platform Investigation Clinique et Accès aux Ressources Biologiques (ICAReB, Institut Pasteur, Paris) through the cohort project Diagmicoll. This protocol was approved by the French Ethical Committee (CPP Ile-de-France I, comprised of Elisabeth Frija-Orvoën, Nadine Forest, Marc Delpech, Michel Hadchouel, Christophe Bardin, Jacques Treton, Janine Taillard, François Dauchy, Cécile Koronkiewcz, Angélique Cozette, Catherine Mazin, Catherine Labrusse-Riou, Antoine Fourment and Pierre Frantz) and the related biospecimen collection was declared to the Research Ministry under the code N° DC 2008-68. 

The animal procedures used to produce monoclonal antibodies were performed according to the European legislation Directive 86/609/EEC [http://ec.europa.eu/food/fs/aw/aw_legislation/scientific/86-609-eec_fr.pdf]. The experimental procedures used in this study caused only short-term or no distress or discomfort. A declaration of distressful experimental procedures will thus not be submitted to the local ethics committee. The prodecures used adhere to the guidelines of the Canadian Council on Animal Care on antibody production (http://www.ccac.ca/Documents/Standards/Guidelines/Antibody_production.pdf). Dr Pierre Lafaye has a personal license (75-61) from the French Ministry of Agriculture and Department of Veterinary Service to perform the animal procedures. Institut Pasteur has an agreement for the laboratory animal containment area (reference B75-15-09). 

To assure ethical animal welfare:

- Buprenorphine was used before each injection;
- The mice were examined every day. Animals exhibiting signs of discomfort received burprenorphine injections;
- The sampling of ascitic fluid was typically undertaken as a final procedure: the mouse was euthanised and then its abdomen was punctured with a needle. The sampling was done before the weight of the animal reached 150% of its initial weight.
- The method of sacrifice for the mice is the carbon dioxide.

### Development and optimization of *S. sonnei* dipstick

The dipstick was developed essentially as previously described [7]. To produce mAbs against the somatic antigen of *S. sonnei*, BALB/c mice were immunized intraperitonally (i.p.) with 10^7^ CFU killed *S. sonnei* bacteria three times at 3-week intervals. Mice eliciting the highest anti-LPS antibody response were given an intravenous boost injection 3 days before being sacrificed for splenic B cell fusion, according to Kohler and Milstein [15]. Hybridoma culture supernatants were screened for antibody (Ab) production by ELISA using LPS purified from *S. sonnei*, as previously described [7,16,17]. Briefly, LPS purified according to Westphal and Jann [18] was used at a concentration of 5 mg/ml in PBS. As secondary Abs, anti-mouse IgG-, IgM peroxidase-labeled conjugate (Sigma-Aldrich) were used at a dilution of 1/5,000. Only the hybridoma cells secreting IgG reacting specifically with LPS homologous to the strain used for immunization, i.e., recognizing serotype-specific determinants on the LPS O-Ag, were selected. The selected hybridomas, representative of the four murine IgG subclasses, were then cloned by limiting dilution, and injected i.p. into histocompatible mice for ascitis production. IgG were precipitated with 50% ammonium sulfate from ascitic fluid, centrifuged, and dialyzed against PBS before being purified using ion-exchange chromatography as previously described [16,17]. Among the available mAbs specific for *S. sonnei*, IgG2b kappa isotype H21-5 was selected for the development of the test.

The Rapid Diagnostic *S. sonnei* (RDSs) test is based on a one-step, vertical-flow immunochromatography using mAb-coupled colloidal gold particles [19]. The colloidal gold particles (40 nm diameter) were conjugated to the H21-5 anti-*S. sonnei* mAb (British Biocell International Cardiff, UK) and lyophilised (A540nm = 2) onto polyester release pads (Accuflow P Schleicher&Shull, Mantes la Ville, France). An automatic thin layer chromatography sampler (CAMAG 5, Muttenz, Switzerland) was used to spray the H21-5 anti-*S. sonnei* mAb at a concentration of 2 μg/cm, as a line on nitrocellulose membrane (Immunopore FP, Whatmann International). In addition, a control capture line was obtained by spraying affinity-purified goat anti-mouse IgG (ICN Biomedical, Aurora, Ohio, USA), on a line higher up on the strip, at a concentration of 1 μg/cm. Cellulose filter paper was used for the wicking and sample pads (Cellulose paper 903, Schleicher & Shull). The immunostrips were then trimmed to a width of 5 mm and stored in a waterproof bag (50 per dissicant bag) at 4°C in Paris (France) or sent to Chile (Facultad de Medicina, Universidad de Chile) and Vietnam (Pasteur Institute Ho Chi Minh City) to be evaluated in clinical studies. 

The test was carried on bacterial strain cultures in broth, on reconstituted stools, on rectal swabs and on stool samples. With bacterial cultures in broth and liquid stools, the test was carried out in 5 ml disposable glass tubes at room temperature with a sample volume of 400 μl. A positive result appears as two strong red lines (upper control line and lower *S. sonnei* 1 LPS positive line), and a negative result as a single upper red control line [7]. *S. sonnei* strain in phase I (ref 1156) was used as a positive control. When testing rectal swab, the faecal swab was immediately plugged 3 minutes in a haemolysis glass tube of 5 ml containing 500 μl of distilled water and then drained inside the tube during 2 minutes; the immunostrip was then introduced in the test tube. The optimal times for the test line and the control line were determined by reading the dipsticks each 30 seconds during preliminary studies on bacterial strain cultures, on reconstituted stools, on rectal swabs and on stool samples.

#### Methodology of the RDSs test evaluation

The evaluations at the bench on strains and reconstituted stools and the evaluation on clinical samples were performed according to the STARD (Standards for Reporting of Diagnostic Accuracy) for new assays [20]. 

### Cut-off, reproducibility, shelf life and specificity

Cut-off, reproducibility, shelf-life and specificity on bacterial cultures were assessed by trained technicians. The cut-off (detection limit) and the range of detectable LPS concentrations was measured using two-fold dilutions (1 000 to 7.5 ng/ml of purified LPS and tenfold dilutions of a S. *sonnei* suspensions (5 x10^3^ to 5 x10^8^ bacteria/ml) using saline, and reconstituted stools (10 g of normal stool without *Shigella* spp suspended in 10 ml of saline). The reproducibility of the cut-off was assessed by testing, ten times simultaneously and using the same batch of RDSs tests on a calibrated suspension of the S. *sonnei* strain ref 1156. To predict the shelf-life of the RDSs test, we used the accelerated stability method that consisted in storing the assays for a time at elevated temperature [19]. The shelf life of the strips in the laboratory was assessed by testing three times per week for 10 weeks after storage at 25°C (air-conditioned room) or at 60°C (incubator). The specificity was assessed using pure cultures of the following bacterial strains: *S. flexneri* serotypes 1a (strain 082429), 1b (strain 085052), 2a (strain 083766), 2b (strain 082831), 3a (strain 084963), 3b (strain 083638), 4 (strain 075519), 4c (strain 08 3649), 6 var Herforshire (strain 083400), 6 var Manchester (strain 080654), Y (strain 075876) and X (strain 08 3347); *S. dysenteriae* serotypes 2 (strain 083092), 3 (strain 081718), 4 (strain 083171), 5 (strain 071059), 6 (strain 087336), 11 (strain 9410434), 12 (strain 080360), 13 (strain 056376) and untypable strain 97-10607, a panel of six wild *S. dysenteriae* 1 strains from Central Africa [38] and five *S. dysenteriae* 1 wild strains from Centre National de Reference des Shigelles at Paris (strains 057331, 100771, 97171, 061306, 061305); *S. boydii* serotypes 1 (strain 07 7695), 2 (strain 08 3129), 3 (strain 07 8186), 4 (strain 08 3330), 5 (strain 599379), 6 (strain 346756), 8 (strain 06 6360), 9 (strain 0541), 10 (strain 081707), 11 (strain 065905), 12 (strain 06 8162), 13 (strain 161055), 14 (strain 08 0226), 15 (strain 04 8291), 17 (strain E3615 53), 18 (strain 078115), 19 (strain 07 5636), 20 (strain 08 2360); *S. sonnei* strains 08 7832, 087159, 087765, 087655 , 087155, 085188, 083857, 062334, 083669, 083141, 062334 (phase 1) and strains 08 7785, 087750, 087866, 087672, 083467 (phase 2) ; *Salmonella enterica typhimurium* (strains 06-2835, 06-2846, 06-2847), *S enteritidis* (strains 06-2841, 06-2844, 06-2851, 06-2852), *S. hadar* (strains 06-2533), *S. brandenburg* (strain 06-2619), *S. heidelberg* (06-2843), *S. oranienburg* (strain 06-2634), *S. risen* (strain 06-2615), *S. stanleyville* (strain 06-2832), *S. typhi* (strain 06-2829), *S. paratyphi* A (strain 06-2633), *S. paratyphi* B (strain 06-2696), *S. meleagridis* (strain 06-2850), *S. stubra* (strain 06-2384), *S. huittingfoss* (strain 06-2391), enteroagregative *Escherichia coli* (strains 55989, JM221, O42, 56390 and 384P), diffusely adherent *E. coli* (strain AL851, AL847, C1845, AL855 and 3043), enterotoxigenic *E. coli* (strains EDL1496, 440TL, Tx-1, E2539-C1, 469), enteropathogenic *E. coli* (strains 135/12 (O55:H-), E6468/62 (O86:H34), 11201 (O125:H6), KK111/1 and F88/6848-2 both O26:H11), *E. coli* O148 (ref CNR E519-66), *Vibrio cholerae* O1 (strains CNRVC960255, 970002, 970014, 970025, 970067, 960325, 970022, 970053, 970055, 970056), *V cholerae* O139 (strains CNRVC 930008, 930381, 930210, 930190), *V. cholerae* non O1 and non O139 (strains CNRVC 930177, 930429, 950689, 950691, 970037, 950769, 910388, 930121, 930297, 930391), *V. alginolyticus* (strain CIP103336), *V. fluvialis* (strains CIP103355, CNRVC356), *V. parahaemolyticus* (strains CIP75.2, CNRVC-030478, CNRVC030479, CNRVC000204, CNRVC000208), *V. furnissii* (strain CIP102972), *V. hollisae* (strain CIP104354), *V. mimicus* (strain 101888), *Aeromonas caviae* (strain CIP76.16), *A. enteropelogenes* (strain CIP104434), *A. hydrophila* (strain CIP76.15), *A. sobria* (strain CIP74.33), *Plesiomonas shigelloides* (strain CIP63.5), *Campylobacter jejuni*, *Yersinia enterocolitica* 1A (6 strains of biotype 1A, 2 strains of biotype 2, 2 strains of biotype 3, and 2 strains of biotype I).

### Evaluation on clinical samples

The RDSs tests were shipped by air mail from France to India, Chile and Vietnam at ambient temperature in grip seal bags.

In Chile, the RDSs test was evaluated on rectal swabs obtained from clinical setting (136 samples from Emergency Room and 88 samples from paediatric department at Hospital Roberto del Río in Santiago, 54 samples from Temuco Regional Hospital, and 64 samples from University of Chile), an area of dysentery endemicity, from December 2008 to April 2009 during the period of high incidence of the disease [21]. For the 342 patients we compared the results obtained with stool cultures for enteropathogenic bacteria and dipsticks performed in a blind way (study) by two different technicians. For each patient, two rectal swabs were collected, one for the stool culture and another to test the dipstick. Between March and April 2009, the RDSs test was also evaluated directly on 51 stool samples collected from patients consulting the emergency Room at Hospital Roberto del Río. 

In Vietnam the evaluation on stools was performed on 60 samples collected and tested in the Paediatric Hospitals I and Pasteur institute in Ho Chi Minh City in 2009 and 2011 (from November 2009 to September 2011). Stool cultures and the RDSs test were performed blindly by two different technicians and the results were then compared. 

In France, 51 non-diarrheic stools from healthy volunteers consulting the Platform ICAReB were tested in 2010. RDTSs were read by two persons and the results were compared at the end of the study. In Chile, Vietnam and France, the dipstick tests were performed with no delay on freshly collected rectal swabs and stools.

In India the RDSs test was evaluated in clinical studies at Chandigarh which is an area of dysentery endemicity involving *Shigella* spp [8,22], from April to November 2012 which is a period of high incidence of the disease. Stool samples were collected from patients admitted to local dispensaries and in district hospitals in Chandigarh. A total of 57 stool samples were collected in sterile screw capped containers and immediately transported to the Medical Laboratory in PGIMER for diagnosis by classical methods by a trained technician. Each of the 57 stools collected were frozen in Cary Blair medium. The 57 frozen stool samples in which the aetiology was known were made available for this evaluation study from the specimen bank of the PGIMER. Stools were encoded. The RDSs tests were performed on defrosted stools by another trained technician.

In the four countries stool samples were cultured immediately after sampling for *Shigella* spp and other enteric bacterial pathogens and analyzed for parasites and viruses by using classical methods with minor modifications according to the laboratories [23]. Suspected colonies resembling *Shigella* were identified biochemically and serotyped by slide agglutination with monovalent O1 sera, according to the International *Enterobacteriaceae* Grouping Subcommittee [24]. 

### Statistics

We calculated the sensitivity (Se), which is the proportion of specimens with the target disorder in which the test result is positive; and the specificity (Sp), which is the proportion of specimens without the target disorder in which the test result is negative. The 95% confidence intervals (CI) for Se and Sp were determined [25]. We also calculated the Cohen’s kappa (κ) statistic [26] to measure concordance between stool culture and the RDSs test in the prospective clinical studies. κ may range from 0 to 1, and a κ value of 0.8 or higher is considered to indicate almost perfect agreement [27]. We also calculated likelihood ratios (LR). The positive LR (LR+ = Se / [1 - Sp]) indicates how many times a positive result is more likely to be observed in specimens with the target disorder than in those without the target disorder. The negative LR (LR- = [1 - Se] / Sp) indicates how many times a negative result is more likely to be observed in specimens with the target disorder than in those without the target disorder. Accuracy increases the more the LR differs from 1. LR+ above 10 and LR- below 0.1 were considered convincing diagnostic evidence [28]. The diagnostic odds ratio (DOR), defined as the ratio of the odds of positive test results in specimens with the target disorder relative to the odds of positive test results in specimens without the target disorder, was calculated as follows [29]: DOR = (Se / [1 - Se]) / ([1 - Sp] / Sp). The DOR does not depend on prevalence and its value ranges from 0 to infinity, with higher values indicating better discriminatory test performance. The positive predictive value (PPV) represents the proportion of test-positive specimens that truly present the target disorder, while the negative predictive value (NPV) represents the proportion of test-negative specimens that truly do not present the target disorder: PPV = (P x Se) / (P x Se) + [(1 - P) x (1 – Sp)] and NPV = (1 - P) x Sp / [(1 - P) x Sp] + [P x (1 - Se)]. P is the prevalence of the target disorder in the population of specimens to which the test is applied. The 95% CI for PPV and NPV were also determined [30].

## Results

The objective of the study was to develop and evaluate a dipstick test for the rapid diagnosis of *S. sonnei* infection using rectal swabs and stool samples at the bedside of the patient. The evaluation was performed firstly by using purified LPS of *S. sonnei* in distilled water and then in reconstituted stools at different concentrations. The dipsticks were then tested on cultures of *S. sonnei* at various concentrations and in reconstituted stools. The dipsticks were also tested with various species of bacteria in cultures, and finally on diarrhoeal stools and rectal swabs.

### Optimal time to read the test

For bacterial cultures, the lower test line appeared in 1 minute and the upper control line appeared 3 minutes later with the two batches. On stools, for positive samples, the strong red lower positive line appeared in 4 minutes and a similar colour appeared on the upper control line 2 minutes later. The optimal delay to read the RDSs test was fixed at 6 minutes. Beyond this time, a weak yellow or purple band was observed on the lower test line. For these reasons it has been stated that RDSs tests must never be interpreted over the defined optimal time for test and control lines. A sample is reported as positive if there is pink to red colour on the test line and on the control line in the optimal time. 

### Cut-off, reproducibility and specificity on bacterial strains

The lower detection threshold of the dipstick for *S. sonnei* LPS was 5 ng/ml in both distilled water and in reconstituted stools. Similar results were obtained using dipsticks stored for 22 days at 56°C. No prozone effect (i. e. no signal detected for high concentrations) was observed by using a range of LPS concentrations extending from 10 ng/ml to 1 mg/ml. In addition, in distilled water and in reconstituted stools containing different concentrations of *S. sonnei*, an unequivocal positive reaction was obtained in 6 minutes with 4 x 10^6^ CFU/ml of *S. sonnei*. These detection limits were reproduced ten times. The specificity of the dipstick was 100% for all bacterial cultures with smooth strains. RDSs tests were always negative with *S. sonnei* strains in phase II. 

### Comparative prospective clinical study

**Figure 1:**
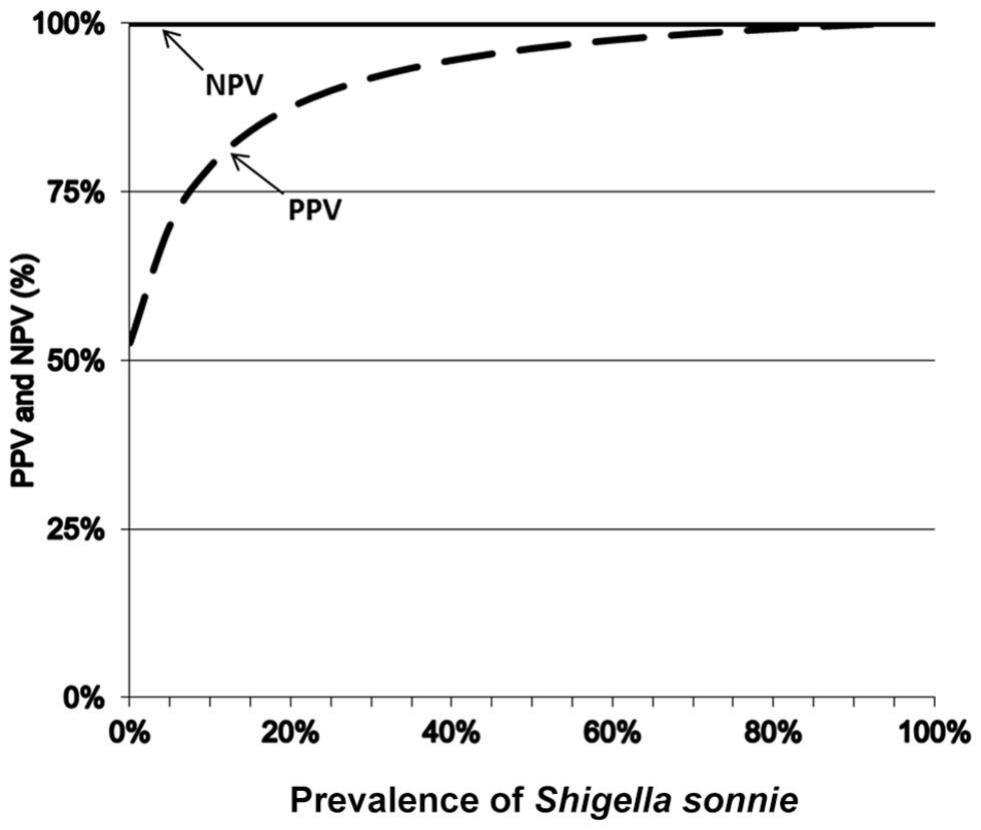
Predictive values (PV) for *Shigella sonnei* diagnosis by using rectal swabs.

In Chile, of the 342 rectal swabs from patients displaying symptoms of acute diarrhea, 47 were both dipstick-and culture-positive, 14 were dipstick-positive but culture-negative, none was dipstick negative but culture-positive, and 281 were negative by both culture and dipstick (Table 1). The test line appears in a mean time of 3 minutes and 40 seconds (range 1 to 5 minutes) and the control line 2 minutes later. For 27 (7.8 %) culture-negative samples a faint yellow or purple band was observed on the lower test line beyond the optimal time. Specificity (281/295) by using rectal swab was therefore 95.3 % (95 % CI 92.9 % - 97.7 %), the sensitivity (47/47) was 100 % (95 % CI – not applicable), positive predictive value (47/61) was 77 % (95 % CI 65 % - 89 %) and negative predictive value (281 / 281) was 100 % (95% CI – not applicable). Stool cultures and RDSs tests on rectal swabs gave concordant results in 95.5 % of cases (47 + 281 / 342) in the comparative studies. The Kappa coefficient obtained in this study was 0.85 ([0.959 – 0.733] / [1 – 0.733]). For the RDSs test, the LR+ was 21.27; it was not possible to calculate LR- and DOR because the Se was 100%. The variations of the PPV and the NPV according to prevalence were determined using the Se and Sp for clinical stool samples (Figure 1).

**Table 1:** Detection of *Shigella sonnei* in 342 rectal swabs by RDSs test *versus* conventional culture.

N° of specimens with *Shigella sonnei* dipstick test result	Bacteriological culture		
	Positive	Negative	Total
Positive	47	14	61
Negative	0	281	281
Total	47	295	342

**Figure 2:**
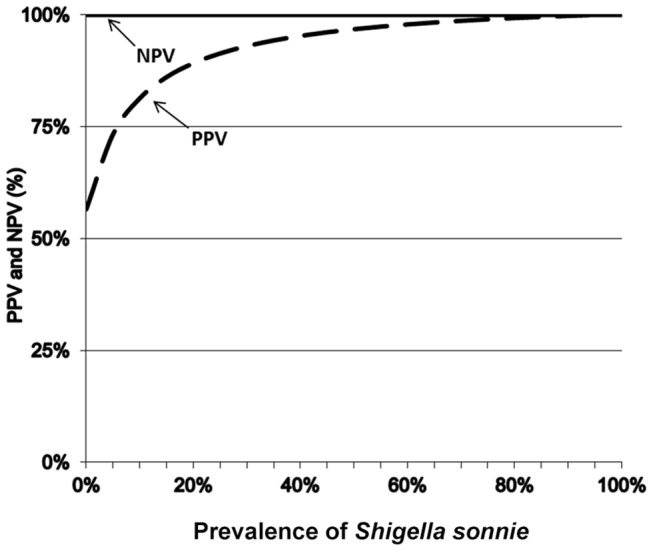
Predictive values (PV) for *Shigella sonnei* diagnosis by using stools.

Of the 219 stool samples (60 from Vietnamese patients, 57 from Indian patients and 51 from Chilean patients displaying symptoms of acute diarrhea, and 51 from healthy French volunteers), 21 were both dipstick-and culture-positive, 8 were dipstick-positive but culture-negative, none was dipstick negative but culture-positive, and 190 were negative by both culture and dipstick (Table 2). Sensitivity (21/21) on the field was therefore 100% (95% CI - not applicable), the specificity (190/198) was 96% (95% CI 92%–98%), positive predictive value (21/29) was 72.4% (95% CI 56.1%–88.6%) and negative predictive value (190/190) 100% (95% CI - not applicable). Stool cultures and RDSs tests on stools gave concordant results in 96.3 % of cases (211/219) in the comparative studies. The Kappa coefficient obtained in this study was 0.82 ([0.963 – 0.797] / [1 – 0.797]). For the RDSs test, the LR+ was 20; it was not possible to calculate LR- and DOR because the Se was 100%. The variations of the PPV and the NPV according to prevalence were determined using the Se and Sp for clinical stool samples (Figure 2).

**Table 2:** Detection of *Shigella sonnei* in 219 direct stools by RDSs test *versus* conventional culture.

N° of specimens with *Shigella sonnei* dipstick test result	Bacteriological culture		
	Positive	Negative	Total
Positive	8 (Chile)	3 (Chile)	29
	6 (Vietnam)	3 (Vietnam)	
	7 (India)	2 (India)	
Negative	0 (Chile)	40 (Chile)	190
	0 (Vietnam)	51 (Vietnam)	
	0 (France)	51 (France)	
	0 (India)	48 (India)	
Total	21	198	219

## Discussion

Because the genome of *Shigella* is highly evolved, it has become a highly specific human pathogen owing to its extensive evolutionary progress involving its repeated gain and/or loss of function compared with *E. coli*. *S. sonnei* becomes the dominant serotype causing shigellosis [5,31].. Furthermore, the review of the situation with regard to shigellosis led to a revision of the World Health Organisation guidelines for the control of bacillary dysentery [32]. The development of a reliable rapid diagnostic assay for improving diagnosis and surveillance is among the main modifications brought to these guidelines [33,34]. Therefore, it is important and essential to develop a simple and reliable test for the sensitive and specific detection of this pathogen.

The conventional culture method currently used for bacterial enteric pathogens remains the gold standard but requires a functioning laboratory and are time-consuming. Currently, *S. sonnei* is isolated from fecal samples using semi-selective media, and the subsequent identification step consists largely of pathogenicity tests and/or molecular typing techniques [35,36,37,38,39,40,41,42,43,44,45,46,47]. Many molecular assays based on ipaH, IS1, tuf, uidA, and the 16S-ITS-23S gene region are generally used for the detection and identification of *Shigella* species, but there have been serious defects in the identification and diagnosis of *S. sonnei* isolates because these assays detect other *Shigella* and enteroinvasive *E. coli* (EIEC) [48,49,50].

Among molecular assays the use of PCR assays can overcome some of the shortcomings of culture methods but the method itself has not yet received global acceptance due to difficulties in its implementation in structures lacking microbiological support. Immunological methods for diagnosis of *Shigella* in stool samples have been studied [51,52,53] but they require a laboratory environment. 

The RDSs test we developed and evaluated has the following characteristics: quick time-to-answer, simple readout, able to be used by minimally trained personnel, the ability to function at 30° C and at high humidity, the ability to be stored for two years without refrigeration, the ability to conduct tests without the need for specific laboratory reagents (only water) or specialized laboratory equipment. This technique is more economical and practical than the traditional methods or the molecular assays. 

Severe and milder forms of shigellosis are developed by patients living in endemic areas. Dysenteric patients have a more severe form of shigellosis with a clinical spectrum ranging from watery diarrhea to diarrhea with mucus and frank bloody diarrhea [54]. Patients who have the most severe form of shigellosis also shed a higher number of microorganisms [54]. A direct relationship between bacterial load (i. e. LPS concentration in stools), detection by culture, and disease severity has also been reported by Thiem et al [13]. Consequently, it is essential to develop an efficient dipstick test displaying a low detection threshold, and detecting the somatic antigen without prozone effect to avoid false-negative results in samples containing high concentrations of *S. sonnei* LPS antigen. We report here such a tool. 

The RDSs test was found to be highly specific when tested on bacterial cultures, with a better detection threshold (4 x 10^6^ CFU/ml of *S. sonnei* and 5 ng/ml of LPS) than dipstick tests developed to diagnose cholera (10^7^ CFU/ml of *V. cholerae* O1 and 50 ng/ml of LPS) [6], *S. dysenteriae* 1 infection (4.9 x 10^6^ CFU/ml of *S. dysenteriae* 1 and 15 ng/ml of LPS) [8] and *S. flexneri* 2a infection (5 x 10^7^ CFU/ml, 20 ng/ml of LPS) [7]. The RDSs detected somatic antigen at a wide range of concentrations, in 5-10 minutes, without prozone effect. Importantly, although the virulent form of *S. sonnei* is unstable due to spontaneous loss of the large virulence plasmid and the ensuing loss of form I O antigen, the RDSs test avoids false negative result because performed directly on fresh or freeze stool samples or rectal swabs.

This RDSs test evaluation on stools and rectal swabs of patients living in four different endemic and non-endemic areas verified its excellent sensitivity and a good specificity. Regarding the problem of decreasing of sensitivity due to humidity previously observed with other dipsticks developed for *S. flexneri* 2a [7], this drawback is now overcome by individual dipstick packaging, making them easily transportable and adapted to the local environmental conditions. 

The reference test – isolation, biochemical and seroagglutination of *Shigella* – which can be done only in the laboratory is specific but lacks sensitivity. This may explain why *S. sonnei* was missed in 22 stools (14 tested with rectal swabs and 8 directly in stools) that were RDSs-positive. Reasons for the low sensitivity of traditional culture methods also include the low number of causative *Shigella* strains in several cases, competition from other commensal microorganisms, and inappropriate changes in ambient temperature and pH during specimen transport [7,8,13,14]. The growth, and thus the detection, of the bacteria is further impaired by the use of antibiotics prior to specimen collection (3 documented cases in this study). Furthermore, regarding these 22 cases, the results are in favor of the RDSs because the test line was clearly positive in the optimal time, no other pathogen was identified and in 14 patients symptoms of invasive enteric infection (fever, white and red blood cells in feces) and severe diarrhea are observed. However, the coproculture remains “indispensable” to complete the diagnosis in particular for determining antibiotic resistance and for characterization of the strains. Given that antimicrobial therapy is recommended for all patients presenting symptoms of dysentery, the clinical significance of a positive rapid diagnostic *S. sonnei* assay is high.

With rectal swabs and on stool samples, the RDSs test and stool cultures gave concordant results in 95.5 % and 96.3 %, respectively, and the kappa coefficient (0.85 and 0.82, respectively) reflected the good agreement. The NPV and the PPV were 77 % and 72.4 %, respectively, even during low prevalence of the disease. The public health implications of this specific and sensitive assay are high in areas where this serotype is rare and in countries where the disease is endemic. This sensitive assay is also valuable to rule out *S. sonnei* diarrhea in an individual patient.

Because these rapid diagnostic tests represent a major breakthrough for individual diagnosis and for surveillance of enteric infections, work is in progress to develop rapid diagnostic tests able to detect multiple pathogens (for *Shigella* spp (generic diagnosis) and the other most prevalent serotypes (*S. dysenteriae* 2 and 3; *S. flexneri* 1b, 2b, 3a, 6b), *Salmonella enterica*, diarrheogenic *Escherichia coli* (EIEC, EPEC, EHEC, EAEC), *Campylobacter* spp, *E. histolytica*, *Giardia lamblia*) or to distinguish between different pathogens and/or strains and subtypes. 

This new diagnosis test, which requires minimal technical skill efficiently complements classical microbiological methods. Late diagnosis is one of the major causes of human death and spread of the disease since it limits the effectiveness of control measures. A highly sensitive test is useful to alert medical authorities to an outbreak of *S. sonnei* diarrhea. At the beginning of an outbreak, critical interventions for severe diarrhea control include improved access to efficient treatment facilities, education to promote good personal hygiene, and improvement of sanitation and safe water supply. Successful interventions depend on early and easy detection of index cases. Such a rapid diagnostic test could also allow better evaluation of the disease burden caused by this organism, therefore improving the evaluation of interventions. 

The challenges ahead are to facilitate access to affordable rapid test (at a price of less than $ 5), to reach sustainable production for the most widespread access and to secure lower-priced rapid tests for countries in the developing world.
